# Editorial

**Published:** 1838-08-29

**Authors:** 


### THE MEDICAL EXAMINER.

PHILADELPHIA, AUGUST 29, 1838.

In the August number of the Medical Journal of the Medical Sciences, is a "Statistical Account of the Cases of Amputation performed at the Pennsylvania Hospital, from January 1st, 1831, to January 1st, 1838," communicated by Dr. George W. Norris, one of the Surgeons. From this paper, it would appear that a much more unfavourable result is likely to attend the operation of amputation, than, without the indisputable evidence of investigations like the present, surgeons, in general, have hitherto been prepared to admit. Hospitals afford the only extended field for the prosecution of exact observation, with any reasonable prospect of ultimate advantage. The opportunities afforded by the most enlarged private practice for the study of any individual disease, must necessarily be limited; and various causes combine to render it otherwise defective. It must, then, be a matter of deep regret, that these institutions are not made subservient to the observation and accumulation of facts, and consequently to the advancement and perfection of science. The basis of knowledge is the collection of well-ascertained facts; and any attempt at generalization, before an extensive acquisition of them has been made, must necessarily be unadvised and premature, and lead to hasty and incorrect conclusions. Persons are too apt to rely for their opinions on their own impressions, instead of depending on a careful notation of facts; and the inevitable consequences, are partial and erroneous views. They will boast of their success in particular diseases; and when reference is made to their case-book, the record usually contradicts the experience of the candidate for therapeutic immortality. The founder of the numerical school of medicine himself acknowledges, that whenever he was tempted to make *a priori* conclusions from recollection, on submitting them to the test of arithmetical analysis, he invariably found them to be erroneous.

We have been induced to make these brief observations from the perusal of Dr. NORRIS' valuable communication, as well as one on the same subject, by Mr. BENJAMIN PHILLIPS, of London, (*Medical Gazette*, June, 1838,) in both of which, by patient inquiry and rigid analysis, results have been obtained respecting a highly interesting and important subject, which will, we suspect, startle not a little many a practical surgeon, who, in the absence of all exact evidence, has been disposed to regard amputation as an operation which, under favourable circumstances, is attended with little hazard. Dr. NORRIS says:

"Contrary to the opinion generally prevalent in this country, amputation, even under favourable circumstances, is frequently followed by fatal results in civil hospitals. In the practice of the Hôtel Dieu, of Paris, it is said that not more than half of the cases prove successful; and I have the authority of M. Hache, a former interne of the hospital of St. Louis, of the same city, for stating, that out of twenty successive amputations made in the year 1833, in that institution, twelve died." (p. 356.)

After a tabular view of the operations performed in the Pennsylvania Hospital for the last seven years, Dr. N. observes:

"Of the above 56 amputations on 55 patients, 24 were primary, of which 14 were cured, and 10 died; 4 of the deaths occurring within the 24 hours immediately following it; 12 were secondary, of which 5 were cured and 7 died; 20* were for the cure of chronic affections, of which 15 were cured and 4 died; 23 of the amputations were of the upper extremity, of which 18 were cured and 5 died; 33 were of the lower extremity, of which 17 were cured and 16 died; 6 were amputations at the joints, of which 4 were cured and 2 died.

Of the 55 patients operated on,

9 were under 20 years of age, of whom 8 were cured and 1 died.
21 between 20 and 30—15 were cured and 7 died.
16 between 30 and 40, 9 " 7 "
9 between 40 and 50, 3 " 6 "

From this resumé of seven years' practice at the Pennsylvania Hospital, it appears,

1st. That amputation is to be regarded as an operation attended with much danger to the life of the individual.

2d. That the chances of success after it are much greater in persons who have been for some time suffering from chronic diseases, than in those who have it done whilst enjoying robust health.

3d. That amputation of the lower extremity is much more fatal than that of the superior member, and

4th. That the danger increases with the age of the individual operated on.

I possess no means for comparing these results with any tabular statements of the success after amputations had in other public institutions, either in this country or in Europe, but the following have been published by some French surgeons as the results of their individual practice. In all of them attempts at union by the first intention, are stated to have been made.

| *Surgeons.* | *No. of Observations.* | *Proportion of Deaths.* |
|---|---|---|
| Dupuytren,* | 29 | 1 in 3 |
| Roux,† | — | 1 in 3 |
| Hyp. Larrey,‡ | 57 | 1 in 6 |
| Dubois,§ | 28 | 1 in 9 |

The unfortunate termination of amputations in France, is attributed, by their surgeons, in the generality of cases, to phlebitis and purulent absorptions. For a long period this termination was thought to be very rare in this country, but post-mortem examination has made known the existence of it in many of the deaths that took place with us, and from all the information I have been able to obtain, I am led to believe that it occurred in the majority of them."—(p. 364–5.)

Mr. PHILLIPS' paper was read before the Royal Medical and Chirurgical Society, in November, 1837. He appears to have been at great pains to obtain correct results; and no doubt can, we think, be entertained of their accuracy, as every precaution seems to have been taken to guard against error. He proposes to show, as the result of his inquiry, that "the mortality after amputation is very great—much greater than is usually believed."

"The amputations included in this inquiry are those of the arm and the forearm, the thigh and the leg. The whole of them have been performed within the last four years in civil hospitals, and in the private practice of hospital surgeons. The gross number of cases is 640; and this number embraces all cases, acute, chronic, and the results of violence, which have occurred in the practice of the persons by whom the returns have been furnished within the period I have named. Of these cases 490 are reported "cured," and 150 died, either in consequence of the operation, or the progress of the disease, to rescue the patient from which, recourse was had to the operation.

"I apprehend that a large number of our professional brethren are unprepared for such a result; I have only met with very few who were at all sensible of the extent of the mortality which occurs.

"Compared with lithotomy, amputation and the ligature of arteries are often, perhaps commonly, held to be unimportant operations; and yet the results show a very great balance in favour of the success of lithotomy."—(p. 459.)

"I have now shown that the mortality succeeding to amputation is very great—23 per cent. I shall therefore proceed to analyse the gross number, and exhibit the proportion furnished by the different countries implicated in the inquiry. They are as follows:

* One of the patients here included suffered double amputation.

* Leçons Orales, tom. iv.

† Mem. et Observ. sur la Réunion.

‡ Sanson. de la Réunion des Plaies.

§ Ibid.

| | Cases. | Deaths. | Per cent. |
|---|---|---|---|
| France, . . . | 203 . . . | 47 or | $23\frac{31}{203}$ |
| Germany, . . | 109 . . . | 26 | $23\frac{93}{109}$ |
| America, . . . | 95 . . . | 24 | $25\frac{5}{19}$ |
| Great Britain, . | 233 . . . | 53 | $22\frac{174}{233}$ |
| | 640 | 150 | $23\frac{7}{16}$ |

"Here is an average number of deaths, amounting to, as near as may be, 23½ per cent. If the several countries be taken separately, we find that France is a fraction below this average; that Germany differs only to the amount of a fraction from France; that America only exceeds the average by a little more than 2 per cent.; and that Great Britain is a fraction below the average."—(p. 460.)

Mr. Phillips then proceeds to examine the causes of this unsuccessful result, and what effect the attempt to procure immediate union of the wound exercises in their production. He thinks that there is a large and well defined class of diseases in which, after amputation, union by the first intention cannot, as at present practised, be so successfully employed, as that in which it is consecutively obtained. This class of diseases, are "diseased articulations, with considerable purulent discharges, necrosis, caries, and extensive old-standing, suppurating surfaces." Of 640 cases constituting the gross number of his tables, 213 were of this kind.

"Of these cases, immediate union was attempted in 117; consecutive union in 96.

Of the 117 cases, 88 only succeeded; the deaths amounted to 29.

Of the 96 cases, in which the treatment was by consecutive union, 76 succeeded; the deaths were 20.

Of these cases, Great Britain furnished 86; the other countries included in the observations, 127.

Of the 86 cases, immediate union was attempted in 60; consecutive in 26, and with the following result: of the 60 cases there were 15 deaths; of the 26 there were 5 deaths.

Of the 127 cases, immediate union was attempted in 57 cases; consecutive in 70.

Of the 57 cases, 14 were unsuccessful, the patients died, and 43 succeeded. Of the 70 cases where consecutive union was employed, there were 15 deaths. The results, therefore, attendant upon the practice of immediate union, are a mortality amounting to 25 per cent.; upon consecutive union, of nearly 21 per cent.

And there is a singular uniformity attendant upon the results of these two modes of practice, as shown by the returns furnished by our own and the other countries; and all are strongly confirmatory of the prudence of avoiding immediate union in this large and well-defined class of diseases.

As I trust this has been made sufficiently evident, it may now be asked, in what way is this increased fatality, which is attendant upon immediate union, to be explained? With respect to a portion of the cases, the explanation is easy. They have been produced by phlebitis and purulent absorptions; pus being found in the lungs, liver, and other situations.

The reason why, in such cases, the tendency to this termination is more frequent than in ordinary cases, is, I apprehend, because the disposition to purulent secretion in the diseased organ still continues; and because immediate union might prevent its evacuation, and so cause constitutional disturbance and absorption.

In a certain number of cases, "visceral congestion" is occasioned by the suppression of the accustomed secretion; diarrhœa supervenes, and the patient dies.

My information is not sufficiently precise to enable me to state the precise proportions of deaths which have been brought about by these several means: under the terms "visceral congestion, abdominal disturbance, and diarrhœa," I have nineteen. Neither am I able to state, with any thing like accuracy, what is the organ in which such disturbance is commonly manifested; but it is evident that the mucous membrane of the intestines, in such cases, is very frequently affected."—(p. 462.)

On this point, Dr. Norris says:

"In none of the operations that I have ever witnessed, has the attempt at immediate reunion obtained a full and complete success. Not unfrequently I have seen a part of the wound united at the first dressing; but in all these cases there has always been a portion of it, other than that at which the ligatures pass out, which has suppurated. In two or three instances, I have known the edges of the skin forming the flap, completely adherent, without being in any degree attached to the bottom of the wound, so that the pus secreted has had no outlet, and the end of the stump has been soft and fluctuating, presenting all the appearances of an abscess."

We trust soon to see other hospital surgeons following the example of Dr. Norris, and publishing the result of amputations in the several institutions to which they are attached.

